# Plant Genetics and Genomics: A Call for Papers

**DOI:** 10.1093/genetics/iyac059

**Published:** 2022-04-27

The journals of the Genetics Society of America, GENETICS and G3: Genes|Genomes|Genetics, are calling for submissions of papers in the area of Plant Genetics and Genomics.

Plant science has played a central role in a broad range of discoveries in Genetics and Genomics, ranging from Mendel’s original transformative studies of inheritance, McClintock’s discovery of mobile genetic elements, to Borlaug’s success in the genetic improvement of plants during the Green Revolution. Plants are also a vital component of the natural biota and the central pillar of human food production and security. A better understanding of both basic and applied plant biology is critical to overcoming a number of challenges currently facing humanity, including combating climate change, world hunger, the production of sustainability energy, and the conservation of biodiversity and ecosystem function.

This series will highlight ongoing advances in Plant Genetics and Genomics by presenting key research findings, new discoveries, and reviews or perspectives. We invite high-quality submissions for all of the journal sections including quantitative traits, gene expression, genome composition, and transmission genetics, but with a special emphasis on plant-environment interaction, genetics and genomics of adaptation, and studies leveraging advanced genomic tools for gene identification and editing to address the issues noted above.

**Series Editors:**

- Thomas Juenger (The University of Texas at Austin)
- James Birchler (University of Missouri)
- Andrea Sweigart (University of Georgia)
- Jianming Yu (Iowa State University)

The Plant Genetics and Genomics Series will launch in Spring 2023. Papers will be collected and navigable across the two journals and promoted on social media and other venues, in a manner similar to other series the journals have published.

Authors are invited to **submit manuscripts by 30 June 2022** and should submit to the journal of choice. Manuscripts will be reviewed and edited through the standard peer review process and according to the usual high standards of the journals. Some manuscripts submitted to GENETICS may be offered a transfer to G3: Genes|Genomes|Genetics instead.

We encourage continued submissions past 30 June 2022 as we intend this series to be an ongoing and growing resource in this area. At submission, please choose the “Plant Genetics and Genomics” article type in the submission systems and mention the series in your cover letter.

Please share with your colleagues, and do not hesitate to contact any of the series editors or the GENETICS (genetics-gsa@thegsajournals.org) and G3: Genes|Genomes|Genetics (g3-gsa@thegsajournals.org) Editorial Office with questions, suggestions, or presubmission inquiries.

